# Associations of maternal perinatal depressive disorders with autism spectrum disorder in offspring: Findings from a data-linkage cohort study

**DOI:** 10.1177/00048674251315641

**Published:** 2025-02-03

**Authors:** Biruk Shalmeno Tusa, Rosa Alati, Kim Betts, Getinet Ayano, Berihun Dachew

**Affiliations:** 1 School of Population Health, Curtin University, Perth, WA, Australia; 2 Department of Epidemiology and Biostatistics, College of Health and Medical Sciences, Haramaya University, Haramaya, Ethiopia; 3 Institute for Social Science Research, The University of Queensland, Brisbane, QLD, Australia; 4 enAble Institute, Curtin University, 1 Kyle Avenue, Bentley, WA, 6102 Australia

**Keywords:** Maternal perinatal depressive disorders, antenatal depressive disorders, postnatal depressive disorders, autism spectrum disorder

## Abstract

**Background:**

There is limited research on the association between maternal depression and autism spectrum disorder, and existing studies face significant limitations, including inadequate control for confounders, reliance on self-reported data, small sample sizes and lack of investigation into mediating factors. This study addresses these gaps by examining the direct relationship and the potential mediating effects of preterm birth, low birth weight and low Apgar scores.

**Methods:**

We analysed linked administrative health data involving 223,068 mother–offspring pairs in New South Wales, Australia. Maternal perinatal depressive disorders and offspring autism spectrum disorder were assessed using the International Classification of Diseases, Tenth Revision, Australian Modification (ICD-10 AM). A generalised linear model was employed to examine the association. The mediation effects of preterm birth, low birth weight and low Apgar scores were assessed through mediation analysis.

**Results:**

After adjusting for a range of potential confounders, offspring of mothers with antenatal, postnatal and overall perinatal depressive disorders had a 61% (risk ratio = 1.61, 95% confidence interval = [1.12, 2.32]), 85% (risk ratio = 1.85, 95% confidence interval = [1.20, 2.86]) and 80% (risk ratio = 1.80, 95% confidence interval = [1.33, 2.43]) higher risk of autism spectrum disorder, respectively. Only about 1.29% and 1.31% of the effect of maternal antenatal depressive disorders on offspring autism spectrum disorder was mediated by preterm birth and low Apgar scores, respectively. Low birth weight had no significant mediating effect on the association.

**Conclusion:**

Maternal perinatal depressive disorders are associated with an increased risk of autism spectrum disorder in offspring. Preterm birth and low Apgar scores were weak mediators of this association. Early intervention strategies that aim to enhance maternal mental health and mitigate the risk of exposed offspring are needed.

## Background

Maternal perinatal depression, occurring through pregnancy to the first year after childbirth, imposes a substantial burden on both mothers and their offspring (Lund and Town, 2016). Recent findings from a meta-analysis reveal that approximately 21% of women endure depression during pregnancy (Yin et al., 2021), while 14% experience postpartum depression in the first year following childbirth (Liu et al., 2022). In Australia, the magnitude of antenatal depression ranges from 10% to 15% (Foundation, 2022), whereas the magnitude of postpartum depressive symptoms ranges from 9% to 14% (Wang et al., 2021).

A range of mental health outcomes, including neurodevelopmental disorders in children, have been consistently linked to maternal perinatal depression (Chithiramohan and Eslick, 2023; Christaki et al., 2022; Dachew et al., 2021, 2023). Among these outcomes, autism spectrum disorder (ASD) is a particularly severe outcome and can affect approximately 1 in 44 children aged 8 years old worldwide (Maenner et al., 2023). ASD is characterised by lifelong challenges in social interaction, communication and participation, with a prevalence rate more than twice as high among boys compared with girls (American Psychiatric Association and Association, 2013; Loomes et al., 2017).

Although genetic predisposition plays a crucial role in the development of ASD, it is widely acknowledged that ASD has a multifaceted aetiology, incorporating genetic, environmental and potentially epigenetic influences (Ghirardi et al., 2018; Kim et al., 2017; Modabbernia et al., 2017; Sandin et al., 2014, 2017). Research suggests that environmental factors contribute to ASD development to varying extents, estimated between 17% and 50%, highlighting the significance of identifying these factors to comprehend the risk of ASD in children (Deng et al., 2015; Kim et al., 2017; Modabbernia et al., 2017; Sandin et al., 2014, 2017). Maternal perinatal depression is recognised as one such risk factor that may contribute to ASD development.

A limited but growing body of epidemiological research has highlighted the association between maternal perinatal depression and the risk of ASD in offspring (Avalos et al., 2023; Chen et al., 2020; Gidaya et al., 2014; Güneş et al., 2023; Hagberg et al., 2017; Hviid et al., 2013; Rai et al., 2013; Say et al., 2016). For example, a recent large prospective cohort study revealed a 2.52 times higher risk of ASD among children born to mothers with depression (Chen et al., 2020). However, existing studies have several limitations. First, some studies have not accounted for a range of important confounding factors, such as socio-economic status (Avalos et al., 2023; Gidaya et al., 2014; Güneş et al., 2023; Hagberg et al., 2017; Say et al., 2016), pregnancy-related complications (Avalos et al., 2023; Chen et al., 2020; Gidaya et al., 2014; Güneş et al., 2023; Hagberg et al., 2017; Hviid et al., 2013; Rai et al., 2013; Say et al., 2016), adverse child outcomes (Avalos et al., 2023; Chen et al., 2020; Gidaya et al., 2014; Güneş et al., 2023; Hagberg et al., 2017; Hviid et al., 2013; Rai et al., 2013; Say et al., 2016) and other maternal psychiatric disorders (Avalos et al., 2023; Chen et al., 2020; Gidaya et al., 2014; Güneş et al., 2023; Say et al., 2016). These factors could potentially influence the observed link between maternal perinatal depression and the risk of ASD. In addition, reliance on self-report screening questionnaires (Gidaya et al., 2014; Güneş et al., 2023; Hagberg et al., 2017; Hviid et al., 2013; Say et al., 2016), rather than more accurate diagnostic assessments of maternal perinatal depression, may limit the precision and reliability of findings. Furthermore, most existing studies have been conducted on relatively small sample sizes (Güneş et al., 2023; Hviid et al., 2013; Say et al., 2016), which could compromise the generalisability and statistical power of the results. Finally, none of the existing studies have examined the potential mediating role of other factors influenced by maternal depression in the association with offspring ASD.

Evidence suggests that maternal depression during pregnancy is a significant risk factor for adverse birth outcomes, such as low birth weight, preterm birth and low Apgar scores (a quick assessment of a newborn’s health, including appearance, pulse, grimace response, activity, and respiration) (Dadi et al., 2020; Sun et al., 2021). This association is thought to arise from the dysregulation of the hypothalamic–pituitary–adrenal (HPA) axis, leading to elevated cortisol levels (Seth et al., 2016), which can negatively impact foetal development. These hormonal disruptions may contribute to adverse birth outcomes, including preterm birth, low birth weight, and compromised foetal well-being, as indicated by low Apgar scores (Field and Diego, 2008). Furthermore, children born with these adverse outcomes are at an increased risk of developing neurodevelopmental and mental health disorders, including ASD. This heightened risk is attributed to disruptions in normal foetal brain development, which may lead to structural and functional abnormalities that increase vulnerability to ASD later in life (Gardener et al., 2011; Laverty et al., 2021; Ma et al., 2022). Therefore, it is plausible that these adverse birth outcomes may mediate any association between maternal antenatal depression and ASD in offspring.

To address the existing gaps in research, our study investigated the risk of ASD in the offspring of mothers diagnosed with depressive disorders, using a large dataset sourced from administrative health records in New South Wales (NSW), Australia. We used robust diagnostic criteria, specifically the International Classification of Diseases, 10th﻿ Revision, Australian Modification (ICD-10 AM), to identify maternal depression and offspring ASD and meticulously adjust for key confounding variables. Moreover, we utilised a mediation analysis to examine whether preterm birth, low birth weight and low Apgar scores mediate the association between maternal antenatal depressive disorder and offspring ASD. The findings from our study hold significant implications for healthcare practices and public health policy, presenting opportunities to enhance maternal mental health and mitigate the risk of ASD in offspring.

## Method

### Study design and population

A retrospective cohort study design was utilised to examine the association between maternal perinatal depressive disorders and the risk of ASD in offspring in the Australian state of NSW.

### Data sources

Mothers and their offspring born between January 2003 and December 2005 and followed until 2018 were connected across three distinct datasets: the NSW Perinatal Data Collection (PDC), the NSW Admitted Patients Data Collection (APDC) and the Mental Health Ambulatory (MH-AMB). These data collections were linked by the NSW Centre for Health Record Linkage (CHeReL). Detailed explanations of the probabilistic linkage and quality assurance methods are available online (linkage CfHR. Master Linkage Key (MLK), 2020).

### Measurement of outcome

Data on offspring with ASD were obtained from NSW records, including primary or secondary diagnoses from APDC and MH-AMB. ASD was defined according to the ICD-10 AM using diagnostic codes F84 to F84.9. These codes encompass a spectrum of conditions within ASD, including pervasive developmental disorders (F84), childhood autism (F84.0), atypical autism (F84.1), Rett’s syndrome (F84.2), other childhood disintegrative disorders (F84.3), overactive disorder associated with mental retardation and stereotyped movements (F84.4), Asperger’s syndrome (F84.5), other pervasive developmental disorders (F84.8) and pervasive developmental disorder unspecified (F84.9).

### Measurement of exposure

Data concerning maternal perinatal depressive disorders were extracted from the APDC and MH-AMB, covering all primary or secondary diagnoses. Diagnosis of maternal perinatal depressive disorders was based on the ICD-10 AM, utilising diagnostic codes F32 through F39. To classify occurrences during the perinatal period, the episode of care containing the diagnosis must occur between the date of conception and the date of delivery (where the conception date was approximated as the delivery date minus the gestational age at delivery) for antenatal depressive disorder. Similarly, for postnatal depressive disorder, the period spans from the date of delivery to 52 weeks post-childbirth.

### Covariates and confounders

Several predetermined covariates and confounder variables were incorporated from the PDC, APDC and MH-AMB datasets into the analysis, aiming to elucidate the association between maternal perinatal depressive disorders and offspring ASD. These factors include socio-economic indicators, sex of the baby, birth order, parity, mode of delivery, antenatal anaemia, antenatal maternal infection, pregnancy-induced hypertension, gestational diabetes, preconception depressive disorder, antenatal anxiety disorder, postnatal anxiety disorder, perinatal bipolar disorder, perinatal schizophrenia disorder, perinatal alcohol use disorder and perinatal substance use disorder (Ayano et al., 2019; Curran et al., 2015; Dachew et al., 2018; Guo et al., 2023; Tadesse et al., 2024; Wu et al., 2017).

### Mediators

In this study, preterm birth, low birth weight and low Apgar scores were examined as mediators. Data on gestational age, birth weight and Apgar scores at 5 minutes were obtained from the PDC datasets. Preterm birth was defined as a live birth occurring before 37 weeks of gestation, while birth weight under 2500 g was categorised as low birth weight. Apgar scores, which assess a baby’s condition shortly after birth, are based on five characteristics: skin colour, pulse, breathing, muscle tone and reflex irritability. Each characteristic is assigned a score between 0 and 2, with a total score ranging from 0 to 10. It is scored at 1 and 5 minutes after birth and Apgar scores below seven at 5 minutes were considered low.

### Statistical analysis

Pearson chi-squared tests were utilised to compare offspring with and without ASD on key socio-economic, maternal and child-related characteristics. To examine the association between maternal perinatal depressive disorders and the risk of ASD in offspring, we employed generalised linear models (GLMs) with a binomial distribution and a log link function, calculating risk ratios (RRs) with 95% confidence intervals (CIs) to quantify the strength and statistical significance of the relationship. Model 1 was unadjusted, Model 2 adjusted for maternal age, socio-economic factors, infant sex, birth order, parity, mode of delivery, antenatal maternal infection, maternal antenatal anaemia, pregnancy-induced hypertension and gestational diabetes, while Model 3 included additional adjustments for maternal mental health and substance use disorders, such as preconception depressive disorder, perinatal anxiety disorder, perinatal bipolar disorder, perinatal schizophrenia, perinatal alcohol use disorder and perinatal substance use disorder. Recognising the high comorbidity and prevalence of depressive and anxiety disorders during the antenatal and postnatal periods, we investigated the combined effect of maternal depressive and anxiety disorders on the risk of offspring ASD. In addition, given the significant comorbidity between ASD and attention-deficit hyperactivity disorder (ADHD; Rong et al., 2021), we examined the impact of maternal perinatal depression on the risk of comorbid ADHD and ASD in offspring.

Furthermore, we conducted a mediation analysis to examine the mediation impact of preterm birth, low birth weight and low Apgar score on the association between maternal antenatal depressive disorder and offspring ASD, using the *paramed* package in Stata 17 (Emsley and Liu, 2013). Initially, we specified two statistical models: (1) the mediator model, which describes the conditional distribution of the preterm birth or low birth weight or low Apgar score given maternal antenatal depression and a set of predetermined covariates and confounders, and (2) the outcome model, which describes the conditional distribution of offspring ASD given maternal antenatal depression preterm birth or low birth weight or low Apgar score, and the same set of predetermined covariates and confounders. The fitted objects from these models served as the main inputs to the mediate function, which computes the estimated natural direct (NDE) and indirect (NIE) effects and marginal total effects (MTE) with standardised β coefficients and 95% CIs derived using a bootstrap option.

The NDE represents the effect of maternal antenatal depressive disorder on offspring ASD independent of each mediator variable. The NIE quantifies the effect of maternal antenatal depressive disorder on offspring ASD mediated by each mediator variable. The MTE represents the sum of the NDE and NIE. To measure the extent of mediation, we estimated the proportion of the association mediated by each mediator using the formula [(NDE × (NIE − 1)) / (NDE × NIE − 1)] (Duko et al., 2024). The 95% CIs of the β coefficients were generated from 1000 bootstrap replicates.

## Results

### Descriptive analysis

Table 1 shows the characteristics of study participants. In our final analysis, we examined a total of 223,068 mother–offspring pairs. Mothers of offspring with ASD were found to be at a higher likelihood of experiencing preconception, antenatal and postnatal depressive disorders, antenatal and postnatal anxiety disorders, perinatal bipolar disorders, schizophrenia, alcohol use disorders, substance use disorders, pregnancy-induced hypertension and gestational diabetes when compared with mothers of offspring without ASD. In addition, more boys than girls were diagnosed with ASD, and offspring diagnosed with ASD were more likely to be born preterm, had low birth weight and presented low Apgar scores compared with those without ASD (Supplemental Table S1).

**Table 1.** Maternal and offspring characteristics by outcome status (*n* = 223,068).

Variables	ASD		Total (%)	*P*-value ^c^
	Yes (%)	No (%)		
Maternal characteristics				
Maternal age				
12–19	124 (8.6)	12,984 (5.9)	13,108 (5.9)	<0.01
20–24	226 (15.6)	26,883 (12.1)	27,109 (12.2)	
25–29	405 (28.1)	61,052 (27.5)	61,457 (27.5)	
30–34	414 (28.7)	75,722 (34.2)	76,136 (34.1)	
⩾35	274 (19.0)	44,963 (20.3)	45,237 (20.3)	
Socio-economic indexes for area				
1st most-disadvantage	464 (32.2)	55,533 (25.1)	55,997 (25.1)	<0.01
2nd most-disadvantage	331 (22.9)	48,529 (21.9)	48,860 (21.9)	
3rd lowest disadvantage	348 (24.1)	54,286 (24.5)	54,634 (24.5)	
4th lowest disadvantage	300 (20.8)	63,089 (28.5)	63,389 (28.5)	
Parity				
Nulliparity	674 (46.7)	91,640 (41.4)	92,314 (41.4)	<0.01
Low multiparity (1–3)	702 (48.7)	122,372 (55.2)	123,074 (55.2)	
Grand multipara (>3)	67 (4.6)	7613 (3.4)	7680 (3.4)	
Mode of delivery				
Normal vaginal	815 (56.5)	136,678 (61.7)	137,493 (61.7)	<0.01
Vacuum extraction	114 (7.9)	15,542 (7.0)	15,656 (7.0)	
Caesarean section	463 (32.1)	61,418 (27.7)	61,881 (27.7)	
Others^a^	51 (3.5)	7987 (3.6)	8038 (3.6)	
Antenatal maternal infection				
Yes	18 (1.2)	1723 (0.8)	1741 (0.8)	0.04
No	1425 (98.8)	219,902 (99.2)	221,327 (99.2)	
Pregnancy-induced hypertension				
Yes	176 (12.2)	17,979 (8.1)	18,155 (8.1)	<0.01
No	1267 (87.8)	203,646 (91.9)	204,913 (91.9)	
Gestational diabetes				
Yes	84 (5.8)	10,923 (4.9)	11,007 (4.9)	<0.01
No	1359 (94.2)	210,702 (95.1)	212,061 (95.1)	
Antenatal anaemia				
Yes	23 (1.6)	4471 (2.0)	4494 (2.0)	0.25
No	1420 (98.4)	217,154 (98.0	218,574 (98.0)	
Preconception depressive disorder				
Yes	17 (1.2)	847 (0.4)	864 (0.4)	<0.01
No	1426 (98.8)	220,778 (99.6)	222,204 (99.6)	
Antenatal depressive disorder				
Yes	34 (2.4)	1906 (0.9)	1940 (0.9)	<0.01
No	1409 (97.6)	219,719 (99.1)	221,128 (99.1)	
Postnatal depressive disorder				
Yes	24 (1.7)	972 (0.4)	996 (0.5)	<0.01
No	1419 (98.3)	220,653 (99.6)	222,072 (99.5)	
Perinatal depressive disorder				
Yes	51 (3.5)	2742 (1.2)	2793 (1.3)	<0.01
No	1392 (96.5)	218,883 (98.8)	220,275 (98.7)	
Antenatal anxiety disorder				
Yes	27 (1.9)	1361 (0.6)	1388 (0.6)	<0.01
No	1416 (98.1)	220,264 (99.4)	221,680 (99.4)	
Postnatal anxiety disorder				
Yes	32 (2.2)	2219 (1.0)	2251 (1.0)	<0.01
No	1411 (97.8)	219,406 (99.0)	221,625 (99.0)	
Perinatal bipolar disorder				
Yes	6 (0.4)	303 (0.1)	309 (0.1)	<0.01
No	1437 (99.6)	221,322 (99.9)	222,759 (99.9)	
Perinatal schizophrenia disorder				
Yes	6 (0.4)	351 (0.2)	357 (0.2)	0.02
No	1437 (99.6)	221,274 (98.8)	222,711 (99.8)	
Perinatal alcohol use disorder				
Yes	13 (0.9)	451 (0.2)	464 (0.2)	<0.01
No	1430 (99.1)	221,174 (99.8)	222,604 (99.8)	
Perinatal substance use disorder^b^				
Yes	63 (4.4)	2615 (1.2)	2678 (1.2)	<0.01
No	1380 (95.6)	219,010 (98.8)	220,390 (98.8)	
Offspring characteristics				
Sex of the baby				
Male	1115 (77.3)	113,928(51.4)	115,043 (51.6)	<0.01
Female	327 (22.7)	107,556 (48.6)	107,883 (48.4)	
Birth order				
First	1418 (98.3)	218,365 (98.5)	219,783 (98.5)	0.41
Second and above	25 (1.7)	3260 (1.5)	3285 (1.5)	
Preterm birth				
Yes	141 (9.8)	13,430 (6.1)	13,571 (6.5)	< 0.01
No	1302 (90.2)	208,195 (93.9)	209,497 (93.5)	
Low birth weight				
Yes	125 (8.7)	11,609 (5.2)	11,734 (5.3)	<0.01
No	1317 (91.3)	209,971 (94.8)	211,288 (94.7)	
Low Apgar score				
Yes	43 (3.0)	2496 (1.1)	2539 (1.1)	<0.01
No	1397 (97.0)	218,943 (98.9)	220,340 (98.9)	

a Others include forceps, vaginal breech and not stated.

b Substance use disorders include tobacco, cannabis, opioids, cocaine, hallucinogens and stimulants.

c Calculated using the Pearson χ^2^ test.

### The risk of ASD in the offspring of mothers with perinatal depressive disorders

Table 2 presents the univariable and multivariable associations between maternal perinatal depressive disorders and offspring ASD. In the univariable analysis (Model 1), offspring of mothers with perinatal depressive disorders had a 2.90-fold higher risk of ASD (RR = 2.90, 95% CI = [2.20, 3.82]) compared with those without such exposure. Specifically, antenatal depressive disorders were associated with a more than two-fold increased risk (RR = 2.76, 95% CI = [1.97, 3.87]), while postnatal depressive disorders were linked to a more than three-fold increased risk (RR = 3.78, 95% CI = [2.54, 5.63]). Adjusting for socio-economic factors and pregnancy-related complications (Model 2) showed sustained elevated risks: antenatal depressive disorders (RR = 2.56, 95% CI = [1.83, 3.58]), postnatal depressive disorders (RR = 3.38, 95% CI = [2.27, 5.04]) and overall perinatal depressive disorders (RR = 2.59, 95% CI = [1.97, 3.42]). Further adjustments for maternal mental health and substance use disorders (Model 3) reduced these risks to 61% (RR = 1.61, 95% CI = [1.12, 2.32]), 85% (RR = 1.85, 95% CI = [1.20, 2.86]) and 80% (RR = 1.80, 95% CI = [1.33, 2.43]) for antenatal, postnatal and overall perinatal depressive disorders, respectively. Perinatal depressive disorders comorbid with anxiety disorders were also associated with a 2.91-fold increased risk of ASD (RR = 2.91, 95% CI = [1.78, 4.74]) (Supplemental Table S2). In addition, maternal perinatal depressive disorder was linked to a 3.93-fold increased risk of comorbid ASD and ADHD in offspring (RR = 3.93, 95% CI = 2.32–6.68) (Supplemental Table S3).

**Table 2.** Univariable and multivariable log-binomial analysis for risk of ASD in offspring of mothers with depression.

Variables	Model 1 RR [95% CI]	*p*-Value	Model 2 RR [95% CI]	*p*-Value	Model 3 RR [95% CI]	*p*-Value
Antenatal depressive disorder						
Yes	2.76 [1.97, 3.87]	<0.01	2.56 [1.83, 3.58]	<0.01	1.61 [1.12, 2.32]^a^	0.01
No	Reference		Reference		Reference	
Postnatal depressive disorder						
Yes	3.78 [2.54, 5.63]	<0.01	3.38 [2.27, 5.04]	<0.01	1.85 [1.20, 2.86]^b^	0.01
No	Reference		Reference		Reference	
Perinatal depressive disorder						
Yes	2.90 [2.20, 3.82]	<0.01	2.59 [1.97, 3.42]	<0.01	1.80 [1.33, 2.43]^c^	<0.01
No	Reference		Reference		Reference	

Model 1 was unadjusted. Model 2 adjusted for maternal age, socio-economic indicators, sex of the baby, birth order, parity, mode of delivery, antenatal maternal infection, antenatal maternal anaemia, pregnancy-induced hypertension and gestational diabetes. Model 3 Adjusted for covariates in Model 2 and maternal mental health and substance use disorders, including preconception depressive disorder, perinatal anxiety disorder, perinatal bipolar disorder, perinatal schizophrenia disorder, perinatal alcohol use disorder and perinatal substance use disorder.

a Further adjusted for postnatal depressive disorder.

b Further adjusted for antenatal depressive disorder, preterm birth, low birth weight and low Apgar score.

c Further adjusted for preterm birth, low birth weight and low Apgar score.

### Mediational effect

**Figure 1.**
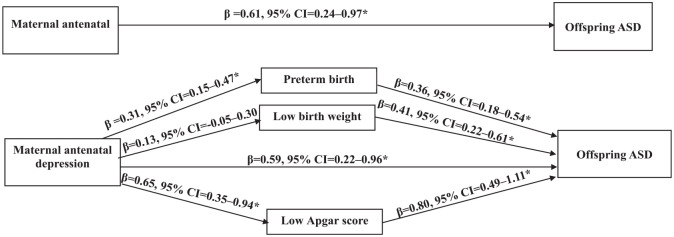
The mediational role of preterm birth, low birth weight and low Apgar, in the association between maternal antenatal depression and offspring ASD. ASD: Autism Spectrum Disorder, Apgar: Appearance Pulse Grimace Activity Respiration, β: standardised beta coefficients, CI: Confidence Interval. The effect values in Figure 1 (a) and (b) were coefficients without and with mediators. All path estimates were adjusted for maternal age, socio-economic indicators, sex of the baby, birth order parity, mode of delivery, antenatal maternal infection, antenatal maternal anaemia, pregnancy-induced hypertension, gestational diabetes preconception depressive disorder, antenatal anxiety disorder, antenatal bipolar disorder, antenatal schizophrenia disorder, antenatal alcohol use disorder, and antenatal substance use disorder. *p < 0.05.

Figure 1 and Table 3 illustrate the mediation role of preterm birth, low birth weight and low Apgar score in the relationship between maternal antenatal depressive disorder and offspring ASD. As shown in Figure 1, maternal antenatal depressive disorder predicted preterm birth (β = 0.31, 95% CI = [0.15, 0.47]) and low Apgar score (β = 0.65, 95% CI = [0.35, 0.94]), but not low birth weight (β = 0.13, 95% CI = [−0.05, 0.30]). When accounting for the effect of preterm birth, low birth weight and low Apgar score, the association between maternal antenatal depressive disorder and offspring ASD remained. However, the effect appeared to be reduced (β = 0.59, 95% CI = [0.22, 0.96]). Table 3 provides regression coefficients for the NDI, NIE and MTE of maternal antenatal depression on offspring ASD. The impact of maternal antenatal depression on offspring ASD was mediated by preterm birth (β = 1.006, 95% CI = [1.002, 1.011]) and low Apgar score (β = 1.006, 95% CI = [1.003, 1.011]), but not by low birth weight (β = 1.002, 95% CI = [0.999, 1.007]). The proportion of the total effect mediated by preterm birth and low Apgar score was only 1.29% and 1.31%, respectively, indicating that this mediation effect was small, about 55 times smaller than the direct effect.

**Table 3.** The mediational role of preterm birth, low birth weight and low Apgar, in the association between maternal antenatal depression and offspring ASD.

Mediator variables	Natural direct effect (NDE) β [95% CI]	Natural indirect effect (NIE) β [95% CI]	Marginal total effect (MTE) β [95% CI]	Proportion of mediated (%)^a^
Preterm birth	1.847 [1.228, 2.594]	1.006 [1.002, 1.011]	1.857 [1.238, 2.592]	1.29
Low birth weight	1.859 [1.242, 2.618]	1.002 [0.999, 1.007]	1.862 [1.230, 2.616]	0.43
Low Apgar	1.823 [1.221, 2.587]	1.006 [1.003, 1.011]	1.834 [1.233, 2.601]	1.31

Models adjusted for maternal age, socio-economic indicators, sex of baby, birth order parity, mode of delivery, antenatal maternal infection, antenatal maternal anaemia, pregnancy-induced hypertension, gestational diabetes preconception depressive disorder, antenatal anxiety disorder, antenatal bipolar disorder, antenatal schizophrenia disorder, antenatal alcohol use disorder and antenatal substance use disorder. The 95% CIs from 1000 nonparametric bootstrap replicates.

a The proportion of the effect mediated by each mediator was computed by [NDE × (NIE − 1)]/(NDE × NIE − 1).

## Discussion

In this large administrative health linkage data, we aimed to assess the impact of maternal perinatal depressive disorders on the risk of offspring ASD and investigate whether preterm birth, low birth weight and low Apgar score mediate this association. Our findings indicate that, after adjusting for various potential confounding factors, offspring of mothers with antenatal, postnatal and overall perinatal depressive disorders had over a 61% increased risk of ASD. We found that the association between maternal antenatal depressive disorders and offspring ASD was weakly mediated by preterm birth and low Apgar scores but not by low birth weight, suggesting that these factors do not fully explain the observed association.

Consistent with our current findings, previous epidemiological studies have also suggested an association between maternal perinatal depression and an increased risk of offspring ASD (Avalos et al., 2023; Chen et al., 2020; Gidaya et al., 2014; Güneş et al., 2023; Hagberg et al., 2017; Hviid et al., 2013; Rai et al., 2013; Say et al., 2016). For example, a large prospective cohort study conducted in Taiwan by Chen et al. (2020) found a more than two-fold increased risk of ASD among offspring of mothers with depression. Similarly, cohort studies in the United States and Denmark reported respective increases of 64% and 82% in the risk of ASD among offspring exposed to maternal antepartum disorder. Furthermore, large case–control studies conducted in the United Kingdom (Hagberg et al., 2017) and Sweden (Rai et al., 2013) have documented a 49% increase in the odds of ASD among offspring of mothers with antenatal depression.

Our findings contrast with studies indicating no association between maternal depression and an increased risk of ASD (Brennan et al., 2023; Castro et al., 2016). For instance, Brennan et al. conducted a retrospective cohort study in the United States, which revealed a null association between maternal antenatal depression and offspring ASD (Brennan et al., 2023). Similarly, a case–control study in the United States reported that children of mothers with antenatal depression showed no increased risk of ASD (Castro et al., 2016). It is important to note that these studies may not have been adequately powered to detect the association between maternal depression and ASD risk in offspring, possibly due to relatively small sample sizes. The discrepancy may also be due to variations in outcome and exposure ascertainment or variability in the selection of potential confounders.

The potential pathways explaining the association between maternal perinatal depression and the risk of ASD in children are still not well explored. However, several mechanisms have been put forward by which maternal perinatal depression may negatively influence the neurodevelopment of offspring and increase the risk of ASD. Epigenetic mechanisms, including changes in DNA methylation, may act as intermediaries connecting maternal antenatal depression to the risk of ASD in offspring (Nemoda and Szyf, 2017). Maternal antenatal depression might alter foetal DNA methylation through the influence of maternal stress hormones, inflammatory cytokines or neurotransmitter levels (Christian et al., 2009; Oberlander et al., 2008; Talge et al., 2007), potentially disturbing the development of the foetal brain and increasing the risk of ASD (Estes and McAllister, 2016; Kinney et al., 2008; Talge et al., 2007).

Alternatively, maternal postpartum depression often coincides with chronic stress and dysregulation of the HPA axis, resulting in heightened cortisol levels (Glynn et al., 2013; Handley et al., 1980). Elevated maternal cortisol during early infancy can disrupt the development of the child’s stress response systems and neural circuits involved in emotional regulation and social behaviour, potentially increasing the risk of ASD (Makris et al., 2021). Moreover, postpartum depression is associated with immune dysregulation and heightened inflammation (Bränn et al., 2020). Maternal inflammatory markers and cytokines can transfer through breast milk, exposing the infant to an inflammatory environment that may impact brain development and elevate the risk of neurodevelopmental disorders, such as ASD (Jiang et al., 2018; Patterson, 2009). In addition, maternal postnatal depression can disrupt the quality of maternal–child interactions, affecting the neurodevelopment of the child and potentially increasing the risk of ASD (Loncarevic et al., 2024; Śliwerski et al., 2020). Women with postnatal depression often struggle to form strong bonds with their children and meet the needs of those at risk of ASD (Śliwerski et al., 2020). This condition can disrupt key caregiving behaviours, such as vocal communication, eye contact and physical touch, which are vital for children’s social, emotional and language development. Children at risk of ASD may be particularly vulnerable to these disruptions, as they require consistent and responsive caregiving to support their development. These difficulties may exacerbate challenges in language and social skills, affecting long-term outcomes.

Furthermore, maternal antenatal depression may elevate the risk of offspring ASD through indirect pathways such as low birth weight, preterm birth and low Apgar score. Maternal antenatal depression can lead to dysregulation of the HPA axis, resulting in increased levels of stress hormones like cortisol (Seth et al., 2016). These elevated stress hormone levels may contribute to adverse birth outcomes such as preterm birth, low birth weight and compromised foetal well-being, as indicated by low Apgar scores (Field and Diego, 2008). Dadi et al.’s umbrella review, drawing from 10 studies, revealed that infants born to mothers experiencing antenatal depression were 40–49% more likely to have low birth weight and to be born prematurely (Dadi et al., 2020). In addition, a systematic review and meta-analysis of prospective cohort studies demonstrated that antenatal depression heightened the risk of a low Apgar score by 91% (Sun et al., 2021).

Preterm birth and low birth weight are also recognised as significant risk factors for neurodevelopmental disorders such as ASD (Gardener et al., 2011; Laverty et al., 2021; Ma et al., 2022). For instance, Laverty et al.’s systematic review and meta-analysis revealed that individuals born preterm were 3.3 times more likely to develop ASD compared with the general population (Laverty et al., 2021). In addition, another systematic review and meta-analysis, pooling data from 28 studies, indicated that infants born with low birth weight had a 63% increased risk of ASD (Ma et al., 2022). These adverse birth outcomes can disrupt the normal development of the foetal brain, resulting in structural and functional abnormalities that heighten vulnerability to ASD later in life. Furthermore, low Apgar scores may signify foetal distress during childbirth, potentially leading to inadequate oxygen supply to the foetal brain. Prenatal hypoxia or ischemia has been linked to altered brain development and an elevated risk of neurodevelopmental disorders, including ASD (Burstyn et al., 2011). Reviews conducted by Gardener et al. further underscored that a low Apgar score is associated with a 67% higher risk of ASD (Gardener et al., 2011). While our study identified associations between preterm birth, low birth weight and low Apgar scores with offspring ASD (Figure 1 and Table 3), our mediational analysis revealed that preterm birth and low Apgar scores accounted for only a minor portion of the overall association between maternal antenatal depressive disorder and offspring ASD and low birth weight did not significantly contribute to this mediational effect, suggesting a direct association between antenatal depression and ASD in offspring.

### Strengths and limitations of the study

The present study stands out due to several notable strengths. First, the substantial sample size enhances its statistical power. Second, the exposure variables (maternal perinatal depression) and the outcome variable (offspring ASD) were measured using the standardised diagnostic tool (ICD-10 AM), ensuring consistency and reliability in assessment. Third, the study counted for a wide array of potential confounders that might influence the association between maternal perinatal depression and offspring ASD. Finally, the study’s inclusion of the mediation effect of low birth weight, preterm birth and low Apgar scores provides a comprehensive understanding of the mechanisms underlying the observed associations.

While our study possesses several strengths, it is crucial to interpret the findings within several limitations. Despite our efforts to account for numerous potential confounders, it is important to acknowledge that we may not have controlled for all relevant variables, leaving the possibility of residual confounding. For instance, we lacked data on maternal autism and autism traits, which are critical confounders in the relationship between maternal perinatal depression and offspring ASD. The high heritability of ASD suggests that some parents of autistic children may exhibit autistic traits (Bolton et al., 1994). Maternal autistic traits, in particular, could predispose women to perinatal depression (Fukui et al., 2023), potentially contributing to the observed association with childhood autism. Paternal depression was also not addressed in our study, though prior epidemiological research has highlighted significant associations between paternal depression and subsequent ASD in offspring (Chen et al., 2020). In addition, we did not adjust for important familial confounding variables, such as genetic and household-level factors, due to the nature of the data. Existing literature strongly suggests that studies on twins indicate median estimated concordance rates ranging from 88% to 95% in identical twins and 31% in fraternal twins (Rosenberg et al., 2009; Taniai et al., 2008). Furthermore, we had no information on maternal depression during child development, which has also been found to be an important environmental factor for the development of developmental outcomes in offspring. Furthermore, our study only enrolled children born between 2003 and 2005 and followed them up to 2018, resulting in a maximum follow-up duration of approximately 15 years. This limited follow-up period may underestimate the prevalence of ASD, particularly as some patients, especially those with mild symptoms, might only be identified during adolescence.

## Conclusion

The findings of this large administrative data-linkage study suggest that maternal perinatal depressive disorders are associated with an elevated risk of offspring ASD. The observed associations were not altered by a range of confounders and mediating factors, highlighting the need for early intervention strategies targeting maternal mental health to mitigate the risks to affected offspring.

## Supplemental Material

sj-docx-1-anp-10.1177_00048674251315641 – Supplemental material for Associations of maternal perinatal depressive disorders with autism spectrum disorder in offspring: Findings from a data-linkage cohort study Supplemental material, sj-docx-1-anp-10.1177_00048674251315641 for Associations of maternal perinatal depressive disorders with autism spectrum disorder in offspring: Findings from a data-linkage cohort study by Biruk Shalmeno Tusa, Rosa Alati, Kim Betts, Getinet Ayano and Berihun Dachew in Australian & New Zealand Journal of Psychiatry

sj-docx-2-anp-10.1177_00048674251315641 – Supplemental material for Associations of maternal perinatal depressive disorders with autism spectrum disorder in offspring: Findings from a data-linkage cohort study Supplemental material, sj-docx-2-anp-10.1177_00048674251315641 for Associations of maternal perinatal depressive disorders with autism spectrum disorder in offspring: Findings from a data-linkage cohort study by Biruk Shalmeno Tusa, Rosa Alati, Kim Betts, Getinet Ayano and Berihun Dachew in Australian & New Zealand Journal of Psychiatry

sj-docx-3-anp-10.1177_00048674251315641 – Supplemental material for Associations of maternal perinatal depressive disorders with autism spectrum disorder in offspring: Findings from a data-linkage cohort study Supplemental material, sj-docx-3-anp-10.1177_00048674251315641 for Associations of maternal perinatal depressive disorders with autism spectrum disorder in offspring: Findings from a data-linkage cohort study by Biruk Shalmeno Tusa, Rosa Alati, Kim Betts, Getinet Ayano and Berihun Dachew in Australian & New Zealand Journal of Psychiatry
