# Supplementary material for: Associations of maternal perinatal depressive disorders with autism spectrum disorder in offspring: Findings from a data-linkage cohort study
Source: Aust N Z J Psychiatry. 2025 Feb 3;59(3):282–92. doi: 10.1177/00048674251315641 (PMC11837418; doi:10.1177/00048674251315641)
Supplement: sj-docx-1-anp-10.1177_00048674251315641 – Supplemental material for Associations of maternal perinatal depressive disorders with autism spectrum disorder in offspring: Findings from a data-linkage cohort study [file sj-docx-1-anp-10.1177_00048674251315641.docx]

**Table S1:** Univariable and multivariable of the risk of ASD in offspring.

| **Variables** | **Unadjusted RR (95% CI)** | **P-value** | **Adjusted RR (95% C)** | **P-value** |
| --- | --- | --- | --- | --- |
| **Maternal characteristics** | | | |  |
| Maternal age |  |  |  |  |
| 12-19 | Reference |  | Reference |  |
| 20-24 | 0.88 (0.71-1.09) | 0.24 | 0.95 (0.76-1.18) | 0.64 |
| 25-29 | 0.69 (0.57-0.85) | <0.01 | 0.81 (0.66-0.99) | 0.04 |
| 30-34 | 0.57 (0.47-0.70) | <0.01 | 0.71 (0.57-0.87) | <0.01 |
| >=35 | 0.64 (0.2-0.79) | <0.01 | 0.77 (0.61-0.97) | 0.02 |
| Socio-Economic Indexes for Area |  |  |  |  |
| 1^st^ most-disadvantage | Reference |  | Reference |  |
| 2^nd^ most-disadvantage | 0.81 (0.71-0.94) | <0.01 | 0.83 (0.72-0.95) | 0.01 |
| 3^rd-^ lowest disadvantage | 0.77 (0.67-0.88) | <0.01 | 0.81 (0.72-0.95) | <0.01 |
| 4^th^ lowest disadvantage | 0.57 (0.49-0.66) | <0.01 | 0.63 (0.54-0.73) | <0.01 |
| Parity |  |  |  |  |
| Nulliparity | Reference |  |  |  |
| Low multiparity (1-3) | 0.79 (0.71-0.87) | <0.01 | 0.86 (0.77-0.97) | 0.01 |
| Grand multipara (>3) | 1.20 (0.94-1.55) | 0.15 | 1.13 (0.87-1.47) | 0.38 |
| Mode of delivery |  |  |  |  |
| Normal vaginal | Reference |  | Reference |  |
| Vacuum extraction | 1.23 (1.01-1.50) | 0.04 | 1.17 (0.96-1.43) | 0.12 |
| Caesarean section | 1.26 (1.13-1.42) | <0.01 | 1.22 (1.08-1.38) | 0.01 |
| Others* | 1.07 (0.81-1.42) | 0.62 | 1.01 (0.76-1.35) | 0.93 |
| Antenatal maternal infection |  |  |  |  |
| No | Reference |  | Reference |  |
| Yes | 1.61 (1.01-2.56) | 0.04 | 1.22 (0.76-1.94) | 0.41 |
| Pregnancy-induced hypertension |  |  |  |  |
| No | Reference |  | Reference |  |
| Yes | 1.57 (1.34-1.84) | <0.01 | 1.40 (1.19-1.64) | <0.01 |
| Gestational diabetes |  |  |  |  |
| No | Reference |  | Reference |  |
| Yes | 1.19 (0.96-1.49) | 0.11 | 1.16 (0.93-1.45) | 0.18 |
| Antenatal anaemia |  |  |  |  |
| No | Reference |  | Reference |  |
| Yes | 0.79 (0.52-1.19) | 0.26 | 0.64 (0.42-0.96) | 0.03 |
| Preconception depressive disorder |  |  |  |  |
| No | Reference |  | Reference |  |
| Yes | 3.07 (1.91-4.93) | <0.01 | 1.68 (1.03-2.73) | 0.04 |
| Perinatal depressive disorder |  |  |  |  |
| No | Reference |  | Reference |  |
| Yes | 2.90 (2.20-3.82) | <0.01 | 1.80 (1.33-2.43) | <0.01 |
| Perinatal anxiety disorder |  |  |  |  |
| No | Reference |  | Reference |  |
| Yes | 2.52 (1.93-3.28) | <0.01 | 1.77 (1.34-2.34) | <0.01 |
| Perinatal bipolar disorder |  |  |  |  |
| No | Reference |  | Reference |  |
| Yes | 3.01 (1.36-6.66) | 0.01 | 1.33 (0.59-2.96) | 0.49 |
| Perinatal schizophrenia disorder |  |  |  |  |
| No | Reference |  | Reference |  |
| Yes | 2.61 (1.18-5.77) | 0.02 | 1.01 (0.45-2.29) | 0.97 |
| Perinatal alcohol use disorder |  |  |  |  |
| No | Reference |  | Reference |  |
| Yes | 4.36 (2.55-7.48) | <0.01 | 1.65 (0.94-2.89) | 0.08 |
| Perinatal substance use disorder** |  |  |  |  |
| No | Reference |  | Reference |  |
| Yes | 3.77 (2.94-4.84) | <0.01 | 2.44 (1.86-3.21) | <0.01 |
| Sex of the baby |  |  |  |  |
| Male | Reference |  | Reference |  |
| Female | 0.31 (0.28-0.35) | <0.01 | 0.32 (0.28-0.36) | <0.01 |
| Birth order |  |  |  |  |
| First | Reference |  | Reference |  |
| Second and above | 1.18 (0.80-1.75) | 0.40 | 0.93 (0.62-1.41) | 0.74 |
| Preterm birth |  |  |  |  |
| No | Reference |  | Reference |  |
| Yes | 1.68 (1.41-1.99) | <0.01 | 1.37 (1.14-1.64) | <0.01 |
| Low birth weight |  |  |  |  |
| No | Reference |  | Reference |  |
| Yes | 1.70 (1.41-2.04) | <0.01 | 1.25 (0.99-1.60) | 0.07 |
| Low Apgar score |  |  |  |  |
| No | Reference |  | Reference |  |
| Yes | 2.68 (1.98-3.62) | <0.01 | 2.00 (1.48-2.71) | <0.01 |
| * Others include forceps, vaginal breech, and not stated; **Substance use disorders include tobacco, cannabis, opioids, cocaine, hallucinogens, and stimulants | | | |  |
