# Supplementary material for: Associations of maternal perinatal depressive disorders with autism spectrum disorder in offspring: Findings from a data-linkage cohort study
Source: Aust N Z J Psychiatry. 2025 Feb 3;59(3):282–92. doi: 10.1177/00048674251315641 (PMC11837418; doi:10.1177/00048674251315641)
Supplement: sj-docx-2-anp-10.1177_00048674251315641 – Supplemental material for Associations of maternal perinatal depressive disorders with autism spectrum disorder in offspring: Findings from a data-linkage cohort study [file sj-docx-2-anp-10.1177_00048674251315641.docx]

**Table S2:** Univariable and multivariable log-binomial analysis for risk of ASD in offspring of mothers with comorbid perinatal depressive and anxiety disorder.

| **Variable** | **Model 1**  **RR (95% CI)** | **P-value** | **Model 2**  **RR (95% CI)** | **P-value** | **Model 3**  **RR (95% CI)** | **P-value** |
| --- | --- | --- | --- | --- | --- | --- |
| **Comorbid perinatal depressive and anxiety disorder** | | | | | | |
| Yes | 4.79 (2.99-7.67) | <0.01 | 4.21 (2.63-6.73) | <0.01 | 2.91 (1.78-4.74) | <0.01 |
| No | Reference |  | Reference |  | Reference |  |
| Model 1 was unadjusted.  Model 2 adjusted for maternal age, socio-economic indicators, sex of the baby, birth order, parity, mode of delivery, preterm birth, low birth weight, low Apgar score, antenatal maternal infection, antenatal maternal anaemia, pregnancy-induced hypertension, and gestational diabetes,  Model 3 Adjusted for covariates in model 2 and maternal mental health and substance use disorders, including preconception depressive disorder, perinatal bipolar disorder, perinatal schizophrenia disorder, perinatal alcohol use disorder, and perinatal substance use disorder. | | | | | | |
